# Review of "Statistical Analysis and Data Display" by Richard M. Heiberger and Burt Holland

**DOI:** 10.1186/1475-925X-4-18

**Published:** 2005-03-18

**Authors:** Francisco Azuaje

**Affiliations:** 1 Computer Science Research Institute, University of Ulster, Jordanstown, Co. Antrim, BT37 0QB, Northern Ireland, UK

Statistical analysis methods and applications have traditionally placed relatively little attention to the graphical visualisation of data and information. Such techniques are fundamental not only for summarising the outcomes originating from statistical analyses, but also for facilitating the detection and assessment of relevant findings. Data-intensive application domains such as medical informatics, biomedical engineering and bioinformatics rely on the application of several statistical analysis and information visualisation techniques to support the generation, validation and integration of hypotheses. This requires an understanding of fundamental principles of data sampling, comparison and classification.

Heiberger and Holland have produced a comprehensive, well-presented review of essential statistical topics. Furthermore, they emphasise the application of graphical display techniques to summarise data and assess analysis results. This book explains why, how and when to apply particular techniques and tools. Another important feature is the incorporation of examples and exercises directly relevant to three leading statistical languages: *S-Plus*, *R *and *SAS*. Executable functions and macros are also provided for several graphical display methods.

After discussing basic statistical concepts, such as probability distributions and hypothesis testing, graphical display techniques are overviewed with an emphasis on scatterplot-based methods. An introductory chapter on inference illustrates how to compare two populations, determine sample sizes and measure goodness of data fit. Chapters dedicated to one-way analysis and multiple comparisons are followed by several chapters on linear, multiple and logistic regression. It also discusses two-way analysis of variance, design of experiments, bivariate and nonparametric statistics. The last section focuses on time series analysis.

The authors, collaborators and publishers made a serious effort to achieve a high quality presentation of text, tables and figures (only black and white). Although the book concentrates on the application of S-Plus, R and SAS, it is a relevant resource to users of other software platforms.

Overall, this is an excellent book for supporting advanced undergraduate courses on statistics or data analysis. Postgraduate students of machine learning and data mining topics may also be greatly benefited from reading most of its chapters. This is a valuable reference source to researchers from these and related areas including medical decision support, clinical engineering and bioinformatics.

